# COVID-19-associated leukoencephalopathy and brain microhemorrhages

**DOI:** 10.1590/0037-8682-0469-2021

**Published:** 2021-09-24

**Authors:** João Vitor Sabino, Eduardo Sellan Lopes Gonçales, Fabiano Reis

**Affiliations:** 1 Universidade Estadual de Campinas, Faculdade de Ciências Médicas, Departamento de Radiologia, Campinas, SP, Brasil.; 2 Universidade Estadual de Campinas, Faculdade de Ciências Médicas, Departamento de Clínica Médica, Campinas, SP, Brasil.

The dissemination and persistence of coronavirus disease 2019 (COVID-19) worldwide have increased recognition of the ability of this disease to cause brain lesions. Herein, we describe a patient with COVID-19-associated leukoencephalopathy with microhemorrhages, a pattern that has been previously described^1^ ^,^ ^2^.

A 62-year-old male patient with hypertension (managed with losartan, 100 mg/day) presented with upper respiratory symptoms that persisted for seven days, along with myalgia. Real-time reverse-transcription polymerase chain reaction testing of a nasopharyngeal swab sample confirmed SARS-CoV-2 infection. The patient was on mechanical ventilation for 37 days, and the lowest recorded blood oxygen saturation level was 90%. The platelet count was normal (311,000/mL).

**FIGURE 1:**
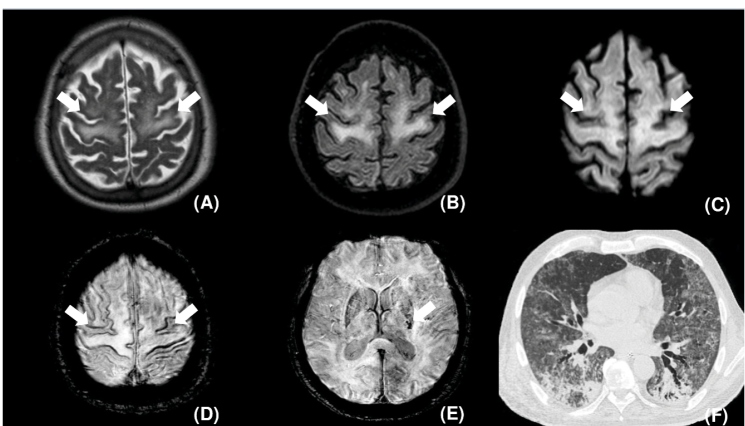
Axial T2 **(A)**, FLAIR **(B)**, and diffusion **(C)** weighted images demonstrating symmetric diffuse hyperintensity (arrows) and mild restricted diffusion (arrows) involving the subcortical white matter in the precentral gyrus and the posterior portions of the superior and middle frontal gyrus. Axial susceptibility-weighted images, **(D)** and **(E),** depict punctate microhemorrhagic foci on the cortical surface of the precentral gyrus, middle frontal gyrus, and superior frontal gyrus (arrows in D) and in the posterior limb of the internal capsule (arrow in E). Chest CT, pulmonary window, axial: diffuse areas of ground-glass opacities and consolidations **(F)**.

A brain magnetic resonance imaging (MRI) scan showed confluent symmetric T2 hyperintensity and restricted diffusion in the subcortical white matter of the precentral gyrus and the centrum semiovale, with small punctate hemorrhagic foci in the posterior limb of the left internal capsule and the subcortical white matter (Figure 1A-E ). Chest computed tomography revealed ground-glass opacities and consolidations in both lungs (Figure 1F ). The patient died three days after MRI.

Although the findings in this case were nonspecific and may be observed in vasculitides^1^ ^,^ ^3^, acute hemorrhagic encephalomyelitis^1^ and delayed post-hypoxic leukoencephalopathy^2^, leukoencephalopathy, and microhemorrhages in critically ill patients with COVID-19 may be related to demyelination, endothelial lesions, and cytokine release syndrome^1^ ^,^ ^2^. These lesions may be viewed as potential late brain complications of COVID-19 in patients with a diminished mental status, and these patients usually have a poor prognosis^1^ ^,^ ^2^.
